# Advances in Nanotechnology for Restorative Dentistry

**DOI:** 10.3390/ma8020717

**Published:** 2015-02-16

**Authors:** Zohaib Khurshid, Muhammad Zafar, Saad Qasim, Sana Shahab, Mustafa Naseem, Ammar AbuReqaiba

**Affiliations:** 1 School of Materials and Metallurgy, University of Birmingham, Birmingham B15 2TT, UK; E-Mail: ZXK338@bham.ac.uk; 2 Department of Restorative Dentistry, College of Dentistry, Taibah University, Al-Madina Al-Munawara 41311, Saudi Arabia; 3 Materials Science and Engineering Department, Kroto Research Institute, University of Sheffield, Sheffield S3 7HQ, UK; E-Mail: s.qasim@sheffield.ac.uk; 4 Department of Dental Materials Science, Sir Syed College of Medical Sciences for Girls, Karachi 75500, Pakistan; E-Mail: drsanashahab@gmail.com; 5 Department of Community and Preventive Dentistry, School of Dentistry, Ziauddin University, Karachi 75600, Pakistan; E-Mail: mustafanasim@hotmail.com; 6 ISF Medical Unit, Qatar and Dental Materials Science, Faculty of Dentistry, the University of Hong Kong, Hong Kong, China; E-Mail: aabureqaiba@isf.gov.qa

**Keywords:** dental materials, nanocomposites, nanodentistry, nanoionomers, nanotubes, dental tissue engineering

## Abstract

Rationalizing has become a new trend in the world of science and technology. Nanotechnology has ascended to become one of the most favorable technologies, and one which will change the application of materials in different fields. The quality of dental biomaterials has been improved by the emergence of nanotechnology. This technology manufactures materials with much better properties or by improving the properties of existing materials. The science of nanotechnology has become the most popular area of research, currently covering a broad range of applications in dentistry. This review describes the basic concept of nanomaterials, recent innovations in nanomaterials and their applications in restorative dentistry. Advances in nanotechnologies are paving the future of dentistry, and there are a plenty of hopes placed on nanomaterials in terms of improving the health care of dental patients.

## 1. Introduction

Nanotechnology is artistic engineering on a scale of less than 100 nm to accomplish desired design, functions and performance of end products. It engages the characterization and control of materials at the atomic or molecular level [1]. At nanoscale, physical, chemical and biological properties are different from the properties at an individual atomic/molecular level and bulk matter [2]. The term nanotechnology was coined by Japanese scientist Dr. Nori Taniguchi in 1974 and was defined as “the processing of separation, consolidation, and deformation of materials by one atom or one molecule” [3]. Long time before the introduction of the term “nanotechnology”, the concept was set up by physicist Richard Feynman in 1959. The idea was entitled as “There’s Plenty of Room at the Bottom” and presented at an American Physical Society meeting at the California Institute of Technology in 1959. Although Feynman did not apply the term “Nanotechnology or Nanosciences” however described the novel process in which scientist can manipulate materials at atomic or molecular levels. The idea of nanotechnology was further probed in depth and promoted by Dr. Drexler and published a book titled “Engines of Creation-The Coming Era of Nanotechnology” around late 1980s. In 1991, the publication by Sumio Lijima “Helical microtubules of graphitic carbon” introduced the concept of nanotubes and boosted nanomaterials research [3,4].

Nanotechnology is one of the most popular areas of current research and has developed in multiple disciplines. There are four main types of materials (metals, polymers, ceramics and composites). Nanomaterials have been developed in all these four categories for practical applications in health care [3]. Due to unique properties, nanomaterials always remain a focus of interest for biomaterials scientists to get benefit for various applications to improve the excellence of life. To date, nanomaterials have been developed and are being used practically for a range of medical applications such as drug delivery, gene delivery, imaging tools, and molecular diagnostics [2,5,6]. Nanomaterials have also been developed for a range of dental applications that are discussed in this review.

## 2. Why Need Nanomaterials in Dentistry?

Despite better understanding of the materials and chemistry, and recent improvements in physical properties, no material has been found that is ideal for any dental application [7]. For example, silver amalgam has been used for dental restoration for more than a century; however, there has been a major concern about mercury toxicity from the amalgam restorations for many years [8,9,10,11,12]. Another major issue is the color of amalgam for aesthetic considerations and alternative materials are being sought to replace [13,14,15]. The composite restorative materials have promising aesthetics however these materials are very technique sensitive and mechanical properties are not as good as of amalgams [16]. Nature has arranged complex biominerals in the best way from the micro to the nano-scale and no one can yet combine biological and physical properties to get ideal structures. In addition, no synthetic material can be intelligent enough to respond to external stimuli and react like nature made tissues [17]. There are a number of possible options to make smart materials for the construction and mimicking of nature (Table 1).

**Table 1:** Options for the production of smart materials for dental applications.

Option	Description
Material Synthesis	Producing synthetic materials matching morphology and properties similar to natural dental tissues.
Biomimetic Approaches	To replace lost dental tissues follow the nature’s principles and producing biomaterials resembling their properties very closely to the replacing tissues.
Tissue Engineering	Use of regenerative medicine and tissue engineering approaches for replacing the lost dental tissues by regenerations.

All these approaches are not possible without the intervention of nanotechnology. For example components required for designing such biomaterials (biomolecules, cells, tissue engineering scaffolds and signal) involve the development of nanomaterials [18,19,20,21,22]. Dental hard tissues (enamel, dentin and cementum) are composed of nanoscale structural units [23] and their mechanical properties such as hardness and elastic modulus may vary from one point to the other [24,25]. Hence, synthetic biomaterials of a similar nature are required to closely match the properties of natural tissues. In addition; there is a huge demand and global market for modern dental materials. For example, the reported global market of resin composite restorative dental materials was €550 million/annum in 2005 [26]. The global market of dental materials is expected to increase rapidly due to multiple factors such as patients’ better awareness of the biomaterials, healthier lifestyle, and increasing population and life expectancy. There is no doubt that the demand of dental biomaterials on a rapid rise and there are no available dental materials having ideal properties for any dental applications. There are plenty of hope surrounding nanomaterials in terms of either developing new materials or significant improvements in the properties of existing materials. The authors have no hesitation to state that the scope of nanomaterials in dentistry is bright and will be helpful to enhance the quality of life patients.

## 3. Approaches in Nanodentistry

**Figure 1:**
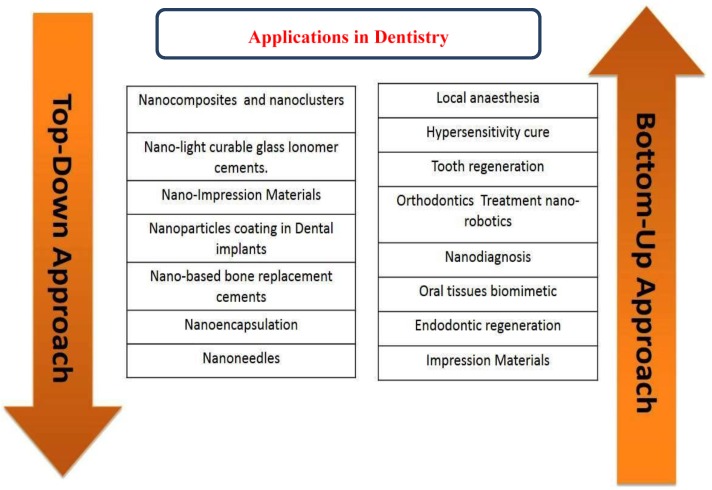
Breakthrough approaches of nanotechnology and their applications in dentistry.

A number of nanotechnology approaches (Figure 1) are being used for a range of practical applications in dentistry [27,28]. There are two key approaches (top down and bottom up) in nanotechnology for creating smaller or better materials and use of smaller constituent into more complex assembling. Top-down approach is based on solid-state processing of materials. Typical examples of top down processes are milling, machining and lithography. The “top-down” approaches such as chemical vapor deposition (CVD), monolithic processing, wet and plasma etching are used to fabricate functional structures at micro and nanoscales [29]. These approaches are successfully used in electronics industry and for coatings of medical implants and stent using CVD technology to enhanced blood flow and biocompatibility [30].

The “bottom-up” approach entangles the fabrication of materials via edifice up particles by harvesting atomic elements [5]. Bottom-up processing is based on extremely organized chemical synthesis and growth of materials. The best example of this approach is present in nature. e.g., repairing of cells, tissues or organ systems and protein synthesis as well [31].

## 4. Applications in Dentistry

There has been remarkable research on nanomaterials in recent years, which has moved it from theoretical foundation to clinical practice. Currently, there is a wide range of nanomaterial’s applications (Table 2) in different subspecialties of dentistry [7,17,19,20,28,32,33,34,35]. As a result of active research for developing new nano-products, the variety of available products for various dental applications is expected to increase remarkably in the near future. 

**Table 2:** Application of Nanotechnology in dentistry with available products.

Discipline	Available Materials
Restorative Dentistry	Ketac™ (3M ESPE, St. Paul, MN, USA), Ketac N100; Nano-ionomers (3M ESPE), Filtek Supreme XT (3M ESPE), Fuji IX GP (GC, Leuven, Belgium), Nano-primer, Premise™ (Kerr/Sybron, Orange, CA, USA), Adper™ Single bond plus Adhesive (3M ESPE), Ceram X™ (DENTSPLY International, Milford, CT, USA).
Regenerative Dentistry and Tissue Engineering	Ostim^®^ (Osartis GmbH, Elsenfeld, Germany), VITOSSO™ (Orthovita-Inc, Malvern, PA, USA), Nano-Bone^®^ (ARTOSS, Rostock, Germany).
Periodontics	Arestin^®^ (Valeant, Bridgewater, MA, USA), Nanogen^®^ (Orthogen, Springfield, IL, USA).
Preventive Dentistry	NanoCare^®^ Gold (Nano-Care, Saarwellingen, Germany).
Orthodontics	Ketac™ N100 Light Curing Nano-Ionomers (3M ESPE), Filtek Supreme Plus Universal (3M ESPE).
Prosthodontics	Nanotech elite H-D plus (Zhermack, Badia Polesine, Italy), GC OPTIGLAZE color^®^ (GC).
Oral Implantology	Nanotite™ Nano-coated implant (BIOMET 3i, Palm Beach Gardens, FL, USA).
Endodontic	AH plus™ (DENTSPLY International), Epiphany (Pentron Clinical Technologies, Wallingford, CT, USA), Guttaflow^®^ (Coltène, Altstätten, Switzerland).

## 5. Nanomaterials for Restorative Dentistry

There has been a drastic evolution in recent years for restorative materials, particularly in tooth colormaterials. Nanotechnologies have been applied for the manufacturing of dental composites (nanocomposites) [35], glass-ionomers cements (nano-ionomers), endodontic sealers and tooth regeneration.

### 5.1. Nanocomposites

The development in the use of nanocomposites patented in response to the persistent and discouraging issues of polymerization shrinkage, strength, microhardness, and wears resistance essential in posterior occlusal applications [36]. Bowen developed the resins [Bisphenol A-Glycidyl Dimethacrylate (Bis-GMA)] and used silane couplers. Around the same era, words like “nano” were coined by the noble laureate Sir Richard Feynman in 1959 [36]. This discovery was a landmark for advances in dental composites. Since then, composite fillings became an essential component of the restorative armamentarium. The last decade has witnessed rapid advances in dental restorative materials including the resin-based composites. The introduction of nanotechnology led to the discovery of nano-filler particles. All efforts were and are being made to achieve considerable advances in physical properties and tackle issues like polymerization shrinkage, wear resistance, micro hardness and achieve patient satisfaction in terms of the aesthetic appearance [16,37].

#### 5.1.1. Composition

Nanocomposites are composed of two or more materials that include a matrix material and nanoscale particles. The matrix should be a biocompatible polymeric, metallic, or ceramic material. In nanocomposites, it is possible to operate the mechanical properties by incorporating secondary nanoparticles to obtain the same characteristic features of natural bone [38]. The properties of nanostructured materials are completely controlled by their synthesis method, processing means and their chemistry [39]. It has been acknowledged that the intrinsic molecular identification of the molecules is governing the formation, morphological development and crystallography of the nanocomposites [40].

#### 5.1.2. Evolution of Organic Resin Matrix

Conventional Resin Based Composites (RBC) were based on organic polymer matrix mainly Bis-GMA and triethylene glycol di-methacrylate (TEGDMA). Due to the hydrogen bonding interactions that are present in between hydroxyl groups and monomer molecules, Bis-GMA becomes very viscous. In order to obtain working viscosity they are mixed with more fluid monomer. In some instances Bis-GMA is combined with tri-ethylene glycol di-methacrylate (TEGDMA) or urethane di-methacrylate (UDMA) or even in some cases by ethoxylatedBisphenol-A-dimethacrylate (Bis-EMA). In order to tackle issues of shrinkage, aging and other environmental factors such as temperature changes and moisture, Bis-GMA is replaced with UDMA or other dimethacrylates [37,41]. Most of the existing methacrylate resin shrinks depending on the number of polymerizable units. This shrinkage is related to monomer percentage. Two methodologies have been used to reduce polymerization shrinkage; either by reducing the reactive sites or using different types of resins. By increasing the filler loading or increasing the molecular weight per reactive group will reduce the density of reactive sites per volume [16].

**Figure 2:**
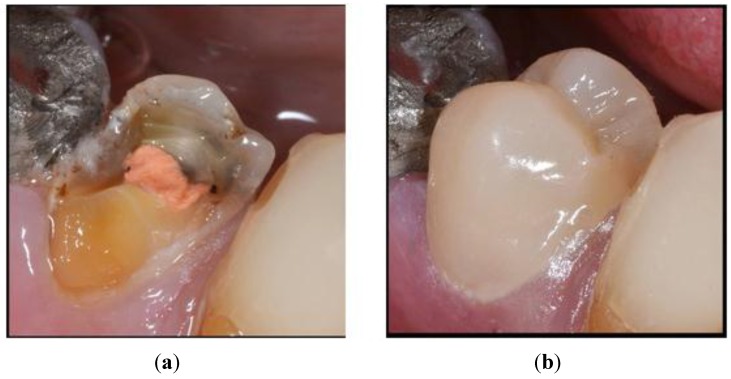
Clinical applications of tooth colored nanocomposite restorative materials (**a**) Root treated and unrestored premolar tooth; (**b**) Crown build up with a post and core using a modern nanocomposite restorative material

The properties of nanocomposites (good translucency, contouring and surface finish) are excellent and can restore lost or damaged dental tissues (Figure 2). Current research is now being focussed on reducing the polymerization shrinking. The addition of using monovinyl methacrylate monomers into dental resin was introduced by Decker, and reported enhanced polymerization kinetics and improved mechanical properties. They were made up of secondary and tertiary functionalities including urethanes, carbonates or cyclic carbonates. They were also referred to as ultra-rapid monomethacrylates. Recently researchers are investigating options of adding acidic functional groups in monomers [42].

#### 5.1.3. A Paradigm Shift of Nano Fillers to Clusters to Hybrids

Carbon nanotubes have superior and exceptional mechanical properties as well as unique bioactivity [43,44]. On the other hand carbon nanotubes lack some essential properties such as hydrophobicity and chemical inertness, which in turn limit their applications [43]. Reinforcing dental composite with carbon nanotubes could help reduce such defects and provoke the advantages gained by excellent mechanical and biological characteristics [45]. Inorganic component of dental composites is made up of filler particles and comprised of quartz or engineered glass particles. Their purposes are to increase strength, modulus of elasticity, reduce polymerization shrinkage and have positive effects on coefficient of thermal expansion and water absorption. Nanohybrid or nanofilled composites are two types of materials referred to the terminology of nanocomposites [46].

**Figure 3:**
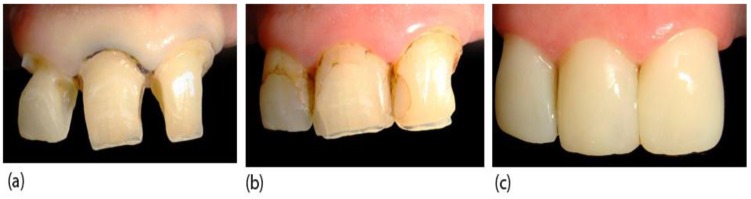
Aesthetic applications of resin nanocomposite restorative materials (**a**) Preoperative labial aspect of defective maxillary anterior segment with recurrent decay and discoloration; (**b**) Composite layering technique adapted to restore decayed tooth structure and midline; (**c**) Postoperative appearance of midline correction using a nanocomposite dental restorative material.

**Figure 4:**
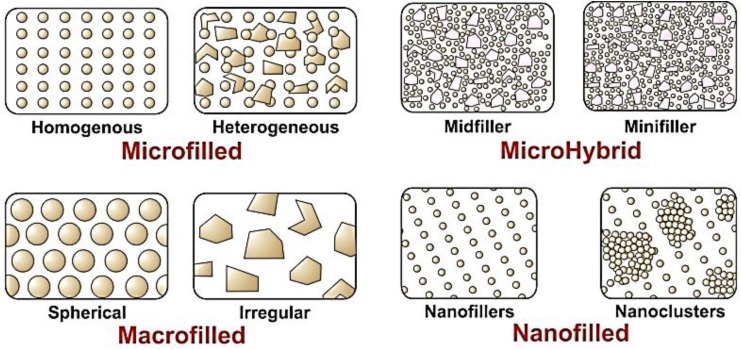
Classification of dental composites on the basis of particle size and structure.

In the process of evolution of dental composites, the alteration of filler size, shape, morphology and loading efficiency still remains a landmark. Various methods adapted to synthesize nanofillers are, flame pyrolysis, flame spray pyrolysis or sol-gel processes. Since the dimensions of these filler particles are below that of visible light, it is impossible for them to either scatter or absorb visible light. This phenomenon plays a key role in getting excellent aesthetic properties and can be used for anterior teeth restorations (Figure 3). Filler loading efficiency can be greater as the size is very small. A direct relationship exist in between the filler loading and surface area of filler particles, this has an effect on the wettability of fillers. What nano based filler particles are doing is they help to improve the continuity in between the macroscopic (40 nm to 0.7 nm) natural tooth structure and nano-sized filler particle. This eventually results in a more natural and advanced interface. Figure 4 depicts the variations in distribution of these nano-filler particles. A homogenous and non-homogenous distribution of nano sized fillers and presence of nanoclusters results in composites with different bulk and surface properties that can be tailor made according to the site of application [36].

Nanofills and nanohybrids are the two different types of more commonly available nanocomposites. Nanofills are dominated by the presence of 1 to 100 nm size particles mainly and nanohybrids are comprised of larger particles ranging from 0.4 to 5 µm, hence they are not truly nanofilled and are called hybrids. For example, NANOSIT™ (Nordiska Dental, Angelholm, Sweden) is a nanohybrid composite comprised of inorganic particles ranging from 7 nm to ≤2000 nm. The uniformity of particle distribution in nanofillers ranges from 5 to 20 nm in a commercially available product 3M ESPE (Filtek™ Supreme Plus, 3M ESPE, St. Paul, MN, USA). Similarly, such interface (hybrid materials) enables increased filler loading and improved adaptability of dental composites. In case of nano-filled composites, it is extremely difficult to control the particle size precisely hence particle size range is used. Various sized nanoparticles facilitate better interface and loading. Comparable results are obtained using nanohybrid and cluster materials.

One of the key purposes of using nanomeric particles is to reduce the particle size than the wavelength of visible light (400 nm to 800 nm). This helps in obtaining highly translucent materials, high surface area to volume ratio and molecular interactions as the polymer size range is usually in the same dimensions. One of the two types of nanomeric particles are nano-agglomerated particles made up of silica or zirconia, surface coated with coupling agent to enhance bonding. Highly filled composites if made using nanomers of the same size have a poor effect on rheological properties [16,37]. In order to overcome this drawback nanoclusters were synthesized by lightly sintering nanomeric oxides resulting in controlled particle size distribution. Nanoclusters act as a bunch of grapes with an average size range of 0.6 µm. These nanoclusters are also surface treated with silane to improve chemical bonding and adhesion with the organic resin matrix. Filtek™ Supreme Plus (3M ESPE) has pioneered the combination of nanoclusters and nanoparticles to obtain better wear resistance. A considerable improvement in the wear properties was observed using the three body wear test performed on an oral wear simulator [16,37].

#### 5.1.4. Coupling Agents

In order to achieve a strong covalent interaction in between the organic matrix and inorganic fillers, coupling agents are used. The coupling agents tend to promote bonding or adhesion between the filler particles and matrix and helping in the transfer of load and stresses. A commonly used coupling agent is gamma methacryloxy propyl trimethoxysilane (MTPS). One side of the coupling agent tends to bond with hydroxyl groups of silica particles and other is copolymerized with polymer matrix [16,37]. Other agents that are added in minor quantity are initiators for light activation, accelerators, pigments for enhancing color and hue. Most commonly used visible initiator is camphoquinone (CQ), its absorption spectrum lies in between 450 and 500 nm wavelength [37].

### 5.2. Nano Glass Ionomers (Nano-Ionomers)

Dental cements are materials in dentistry that are used frequently. There is no universally accepted cement that fulfills all applications; there are a variety of cements whose properties and manipulation lead them to be an appropriate choice for a given application. The retention of restorations on prepared teeth is a major function of dental cements. Long-term cementation is required for permanent restorations such as crowns and bridges. Strong cements, such as compomer, glass ionomers, hybrid ionomers, zinc phosphate, zinc poly-carboxylate, or resin-based cement, are used for the long term cementation [47,48].

**Figure 5:**
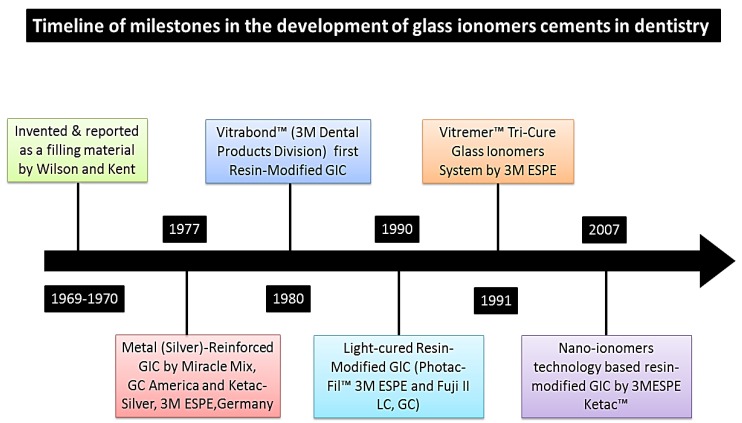
Timeline of milestone in the development of glass ionomer cements and nano-ionomers for dental restorations.

Glass ionomers were introduced by Wilson and Kent in the 1970s as dental filling material [49]. The timeline of milestone in the development of glass ionomers cement in dentistry is described in Figure 5. Glass ionomers are supplied in powder and liquid form and work on the principle of acid base reaction. The powder is composed of mainly fluoro-alumino-silicate (FAS) glass particles and ions such as strontium, calcium and lanthanum. The liquid is a copolymer of acrylic acid and itaconic acid or maleic acid and supplied as a viscous fluid. Acid base reaction takes place while powder and liquid are mixed and initial set takes place in 3–4 min [50]. Glass ionomers are being used widely because of its excellent properties such as chemical bonding to the tooth structure, biocompatibility and fluoride release. On the other hand, there are a number of shortcomings for this group of materials such as poor aesthetic, prolonged setting reaction, compromised mechanical properties and weaker bond strength [51]. In order to improve the properties and to overcome these shortcomings, active research is in progress, such as in the addition of cellulose fibers, hydroxyapatite and fluoroapatite and nanotechnologies [52].

More recently, nanotechnologies have been applied to the resin modified glass ionomers in the form of nanoparticles (nanomers) and nanoclusters in fluoroaluminosilicate (FAS) glass. These nanoionomers (Ketac™ Nano; 3M ESPE) have been available for clinical use since 2007 [53]. The addition of nanoparticles resulted in the aesthetic improvement of the final restoration and polishablilty [54]. It is important to mention that fluoride release property is not affected by addition of nanoparticles due to the high surface area. In a recent study, the Knoop hardness of Ketac™ Nano was observed to be lower (39 KHN) than resin modified (Vitremer) glass ionomers (69.9 KHN). The Knoop hardness of Ketac™ Nano (48 KHN) is not fulfilling the American Dental Association (ADA) specifications for restoration hardness; hence cannot be recommended for high stress bearing areas. The manufacturer (3M ESPE) has recommended its use for class I, class III, class V, under the composite and primary teeth. Tancan, *et al.* reported shear bond strength (SBS) of nanoionomer and nanocomposites is good as compare with conventional GICs orthodontic bracket bonding [55].

Another development is the introduction of a new nanomaterial called Equia^®^ System. Inorganic silica nanofillers (40 nm size) are dispersed in liquid and reinforce the resulting polymer matrix. Adding 15 wt. % silica nanofillers resulted in good wear resistance and reduction in the initial setting time. Due to better resistance dissolution, disintegration and wear, these materials were observed to maintain polished surface for a longer period of time [56]. There is an enhancement of optical properties and translucency compared to conventional glass ionomers and aesthetic appearance is improved remarkably and claimed as good as of natural teeth [56]. Friedl *et al.* present a retrospective study on new glass ionomers cements to evaluate their performance and concluded Equia^®^ System is good for posterior filling materials [57].

The effects of additives such as nanoscale hydroxyapatite (HA) and fluoroapatite (FA) on properties of glass ionomers is a hot topic for researchers [34]. Hydroxyapatite crystals are well known for their biocompatibility and have made a major contribution in the chemical structure of natural enamel and dentin. The bond strength of resin modified glass ionomers to the tooth structure was measured 8–12 MPa [58]. The bond strength was increased with addition of micro HA (5–10 µm) and whereas Nano-HA (100–150 nm) further improved the bond strength. This improved strength is probably related to the availability of a higher surface area for bonding to tooth structure in case of Nano-HA. This high surface area also increases the surface finish and solubility of Nano-HA that helps filling the demineralizing micro-pores in the tooth structure [54]. Similarly, Nano-HA and Nano-FA prepared using ethanol based sol-gel methods were added to Fuji II glass ionomers [54]. The modified nano-filled glass ionomers exhibited better mechanical properties such as compressive strength, diametral tensile strength and biaxial flexural strength. Modification of existing glass ionomers using nanomaterials is an active area of current research. A number of other materials such as alumina, alumina/titania, zirconia and yttria stabilized zirconia. There are very high expectations from this on-going research on nanomaterials in relation to glass ionomers. As a result, it can be hoped that there will be an addition of new dental materials in the near future or the properties of existing glass ionomers will be improved significantly [59].

### 5.3. Endodontic Sealer

The applications of nanotechnology are not limited to filling materials but have been extended to endodontic applications. A bioceramic based nanomaterials (EndoSequence BC sealer) composed of calcium silicate, calcium phosphate, calcium hydroxide, zirconia and a thickening agent, has been developed recently. Nanoparticles have improved the handling and physical properties. During the hydration reaction in the root canal, a nanocomposites structure of calcium silicate and hydroxyapatite is formed. The hydration reaction and setting time is affected by the availability of water [60] and setting time may be prolonged in overly dried canals. Nano sized particles facilitate delivery of material from 0.012 capillary needle and adopt to irregular dentin surfaces. It sets hard in a matter of a few hours providing excellent seal and dimensional stability. Upon setting, it forms of hydroxyapatite; providing biocompatible and bioactivity. The highly alkaline pH (12.8) gives antimicrobial properties as well [60,61].

Another example is a silicon based sealer (Gutta-Flow Sealer) with an addition of gutta-percha powder and silver nanoparticles. This material is available in the form of uni-dose capsule that can be mixed and injected [61]. This nano-sealer has good biocompatibility, dimensionally stable and sets within half an hour. This material has been reported to improve the sealing capability and better resistance to bacterial penetration. For infection point of view, the antibacterial activity of endodontic sealers can be very beneficial. Recently, antibacterial quaternary ammonium polyethyleneimine (QPEI) nanoparticles have been incorporated into existing sealers such as AH plus, Epiphany and Guttaflow [62]. Resin composites containing QPEI nanoparticles resulted in prolonged antibacterial activity without compromising the mechanical properties [63]. In order to obtain similar antibacterial effect in endodontic sealers, 0-2 wt% QPEI nanoparticles were added in to the commercially available sealers [62]. The addition of QPEI nanoparticles is very stable, leaching no byproducts in the surrounding and there was no effect on the biocompatibility; however, the antibacterial properties remained excellent [62]. 

### 5.4. Nanomaterials for Dental Tissue Regeneration

The applications of nanoscale scaffold materials for tooth tissue regeneration are well established. For pulp regeneration, pulp stem cells were purified in the laboratory and grown in sheets on scaffolds. The scaffolds used were composed of nanofibers of biodegradable collagen type I or fibronectin [64,65]. Self-assembling polypeptide hydrogels have been used for pulp tissue regeneration. There is formation of a nanofiber mesh that supported the growing cells [66]. Puramatrix (containing amino acids repeats of alanine, arginine and aspartate) has been proven to enhance cell growth [67]. Natural silk based nanomaterials are being used for various tissue regeneration applications and have promising scope for dental applications [68]. Injectable self-assembly collagen I scaffold loaded with exfoliated teeth stem cells resulted in the formation of pulp like tissue and functional odontoblasts [69]. Collagen type I is the most abundant fibrous protein found in the form of nanofibers in dentin (~80%–90% of organic matrix) and bone [70]. Odontogenic differentiation and mineralization was promoted in the presence of type I collagen scaffolds [71,72]. The tissue regeneration approached are not in practical implementation at present, however further research is expected to overcome the challenges to fancy tissue engineering products available for clinical applications in near future.

## 6. Conclusions

There is no doubt that the demand of dental biomaterials on a rapid rise and there are no available dental materials with ideal properties for any dental applications. There are plenty of hopes from nanomaterials in terms of either developing new materials or significant improvements in the properties of existing materials. Advances in nanotechnologies are paving the future of dentistry. The authors have no hesitation to state that the scope of nanomaterials in dentistry is bright and will be helpful to enhance the quality of life of patients. For example, concept of using nano-robots or denti-robots will fight bacteria and harbor them within the oral flora, diagnosis and treatment time span to reduce from months to days or days to hours will be the aftermath of scientific research in nano-dentistry. All this will become a possibility within the time span of 10–20 years. This is an area of very active research all around the globe involving a lot of research funding. It can be expected in future that the science of dental materials may change significantly with better understanding and the introduction of new nano-biomaterials. 
